# Identification of anti-citrullinated osteopontin antibodies and increased inflammatory response by enhancement of osteopontin binding to fibroblast-like synoviocytes in rheumatoid arthritis

**DOI:** 10.1186/s13075-023-03007-9

**Published:** 2023-02-17

**Authors:** Akio Umemoto, Takeshi Kuwada, Koichi Murata, Masahiro Shiokawa, Sakiko Ota, Yoshiki Murotani, Akihiro Itamoto, Kohei Nishitani, Hiroyuki Yoshitomi, Takayuki Fujii, Akira Onishi, Hideo Onizawa, Kosaku Murakami, Masao Tanaka, Hiromu Ito, Hiroshi Seno, Akio Morinobu, Shuichi Matsuda

**Affiliations:** 1 Department of Orthopaedic Surgery, Kyoto University Graduate School of Medicine, Sakyo, Kyoto, 606-8507 Japan; 2 Department of Gastroenterology and Hepatology, Kyoto University Graduate School of Medicine, Kyoto, 606-8507 Japan; 3 Department of Advanced Medicine for Rheumatic Diseases, Kyoto University Graduate School of Medicine, 54 Kawahara-Cho, Shogoin, Sakyo, Kyoto, 606-8507 Japan; 4 Department of Immunology, Kyoto University Graduate School of Medicine, Sakyo, Kyoto, 606-8501 Japan; 5 Center for Cancer Immunotherapy and Immunobiology, Kyoto University Graduate School of Medicine, Sakyo, Kyoto, 606-8507 Japan; 6 Department of Rheumatology and Clinical Immunology, Kyoto University Graduate School of Medicine, Sakyo, Kyoto, 606-8507 Japan

**Keywords:** Rheumatoid arthritis, Anti-citrullinated protein antibody, Osteopontin, Citrullination, Integrin

## Abstract

**Background:**

Anti-citrullinated protein/peptide antibodies (ACPAs) are present in patients at onset and have important pathogenic roles during the course of rheumatoid arthritis (RA). The characteristics of several molecules recognized by ACPA have been studied in RA, but the positivity rate of autoantibodies against each antigen is not high, and the pathogenic mechanism of each antibody is not fully understood. We investigated the role of anti-citrullinated osteopontin (anti-cit-OPN) antibodies in RA pathogenesis.

**Methods:**

Enzyme-linked immunosorbent assays on RA patients’ sera were used to detect autoantibodies against OPN. Fibroblast-like synoviocytes (FLS) isolated from RA patients were used to test the binding activity and inflammatory response of OPN mediated by anti-cit-OPN antibodies, and their effect was tested using an inflammatory arthritis mouse model immunized with cit-OPN. Anti-cit-OPN antibody positivity and clinical characteristics were investigated in the patients as well.

**Results:**

Using sera from 224 RA patients, anti-cit-OPN antibodies were positive in approximately 44% of RA patients, while approximately 78% of patients were positive for the cyclic citrullinated peptide (CCP2) assay. IgG from patients with anti-cit-OPN antibody increased the binding activity of OPN to FLSs, which further increased matrix metalloproteinase and interleukin-6 production in TNF-stimulated FLSs. Mice immunized with cit-OPN antibodies experienced severe arthritis. Anti-cit-OPN antibodies in RA patients decreased the drug survival rate of tumor necrosis factor (TNF) inhibitors, while it did not decrease that of CTLA4-Ig.

**Conclusions:**

Anti-cit-OPN antibodies were detected in patients with RA. IgG from patients with anti-cit-OPN antibodies aggravated RA, and anti-cit-OPN antibody was a marker of reduced the survival rate of TNF inhibitors in RA patients.

**Supplementary Information:**

The online version contains supplementary material available at 10.1186/s13075-023-03007-9.

## Introduction

Rheumatoid arthritis (RA) is characterized by synovial inflammation and destruction of the joint cartilage and bone and mediated by chronic proinflammatory cytokines and matrix metalloproteinases [1]. Identification of RA at initial presentation and initiation of treatment at early stage is important for preventing the development of bone erosion and retarding the progression of the disease [2, 3].

Anti-citrullinated protein/peptide antibodies (ACPAs) are sensitive and highly specific biomarkers for the diagnosis of RA that were present years before the onset of clinical RA [4, 5]. ACPA positivity has also been used to predict severe erosive disease. Commercial assays use synthetic cyclic citrullinated peptides (CCP) that are structurally different from the naturally occurring proteins in the joint. While such assays are highly efficient at diagnosing RA, they are of limited use in analyzing the disease pathogenesis mechanism [6, 7].

Though more than 20 molecules recognized by ACPA have been studied [8]; only a few (e.g., citrullinated fibrinogen (cit-Fgn), citrullinated vimentin (cit-Vim), citrullinated α-enolase peptide 1, and citrullinated tenascin-C (cit-TNC)) have been proven to be present in the joint, studied in large cohorts, with successful epitope-mapping carried out and their antigen specificity confirmed [9]. The diagnostic sensitivity of each of these peptides (30–50%) is lower than that of anti-CCP2 assay [9, 10].

Osteopontin (OPN) is a transformation-associated phosphoprotein belonging to the small integrin-binding ligand N-linked glycoprotein (SIBLING) family encoded by the SPP1 gene [11]. It is synthesized in a variety of tissues and cells, including osteoclasts, chondrocytes, synoviocytes, macrophages, lymphocytes, and vascular smooth muscle cells, and is secreted into body fluids. It has a specific arginine-glycine-aspartate (RGD) sequence; thus, it can be recognized and bound to the corresponding integrin on the cell surface, making it important for cell adhesion and migration [11, 12].

Numerous studies have indicated an increased expression of OPN, including cleaved OPN, and its relationship with RA pathology [13]. OPN expression is upregulated in the RA synovial lining and cartilage interface, invading the synovium, plasma, and synovial fluid [11]. OPN regulates Th17 differentiation, CD14 + monocyte migration, cytokine production of fibroblast-like synoviocytes (FLS) through interaction with B lymphocytes, and neutrophil viability in RA [11]. Thus, it is a potential therapeutic target for RA treatment.

Anti-OPN antibodies were detected in 15% of RA, and citrullination was suggested to increase the sensitivity [14, 15]. OPN is considered to be an autoantigen in RA. However, it remains unclear whether anti-citrullinated OPN (cit-OPN) antibodies are involved in RA pathology. Here, we investigated the involvement of the anti-cit-OPN antibodies in RA pathology and their role as biomarkers.

## Materials and methods

### Reagents and antibodies

Recombinant osteopontin was purchased from R&D Systems (Minneapolis, MN, USA, #1433-OP-050/CF), ACROBiosystems (Newark, DE, #OPN-H5227), and Abcam (Cambridge, UK, #ab92964, #ab281819). Fibronectin, tenascin-C, bone sialoprotein, and vimentin were purchased from R&D Systems (Minneapolis, MN, USA). a-enolase and type II collagen were purchased from Abcam (Cambridge, UK). Fibrinogen was purchased from the Fujifilm Wako Pure Chemical Corporation (Osaka, Japan). Antibodies against total focal adhesion kinase (FAK) and phosphorylated FAK were purchased from Cell Signaling Technology (Danvers, MA, USA, #3285 and #8556). Recombinant human tumor necrosis factor (TNF) was purchased from Peprotech (Cranbury, NJ, USA, #300-01A). The secondary antibody used for immunoblot analysis was peroxidase-conjugated anti-rabbit IgG (Agilent, Santa Clara, CA, USA; #P0399).

### Patients

Sera and/or synovial tissue were obtained from RA patients, non-RA patients, or healthy donors according to a protocol approved by the Ethics Committee of the Graduate School of Medicine, Kyoto University. Written informed consent was obtained from all participants. All serum samples were stored at − 80 °C until assayed. All patients with RA met the 1987 American College of Rheumatology revised criteria [16] or the 2010 American College of Rheumatology (ACR)/European League Against Rheumatism (EULAR) criteria [17].

As a training group, 30 patients with RA were selected from the Kyoto University Rheumatoid Arthritis Management Alliance (KURAMA) cohort. Of these, 29 patients had high Disease Activity Score-28 for Rheumatoid Arthritis with erythrocyte sedimentation rate (DAS28-ESR) and were positive for C- reactive protein (CRP), and 25 of them were positive for the anti-CCP antibody. One patient in remission was included in this study. Sera from seven healthy donors were used as controls. For the validation study, antibody titres of cit-OPN were measured in patients who participated in an annual survey of the KURAMA cohort for three consecutive years starting in 2016, which consisted of 224 RA patients. Control serum was obtained from 91 donors, consisting of 38 patients with osteoarthritis and 53 healthy controls, who were referred to the Kyoto University Center for Rheumatic Diseases. Autoantibody titer of 4.5 U/mL or higher for anti-CCP antibodies and ≥ 15 IU/mL for RF were defined as positive.

### Enzyme-linked immunosorbent assay (ELISA)

To citrullinate the recombinant proteins, proteins at a final concentration of 100 mg/ml were incubated with 5 units/ml of rabbit PAD (P1584, Sigma-Aldrich, St. Louis, MO) in working buffer (100 mM Tris–HCl, 5 mM CaCl2, pH 7.4) for 18 h at 37 °C [18]. Rabbit peptidyl arginine deiminase (PAD) is a counterpart of human PADI2 and is highly homologous to human PADI4. For the detection of serum IgG antibodies, the ELISA Starter Accessory kit (E101; Bethyl Laboratories, Montgomery, TX, USA) was used according to the manufacturer’s instructions. Briefly, microtiter plates were coated with 100 mL of 2 mg/mL recombinant protein, incubated at room temperature for 1 h, blocked, and incubated with 100 mL of diluted patient serum (1:100) for 60 min. After washing, the plate was incubated with 100 mL of horseradish peroxidase (HRP)-conjugated rabbit anti-human IgG antibody (1:50,000; ab6759; Abcam, Cambridge, UK) at room temperature for 60 min. After washing, bound reactants were detected by incubation with 3,3′3Cambrtetramethylbenzidine for 25 min. The absorbance was measured at 450 nm using a microplate reader (iMark™ Microplate Absorbance Reader, Bio-Rad Laboratories, Hercules, California, USA) [19].

### Preparation of human IgG

Ab-Rapid SpiN (P-013, ProteNova, Higashikagawa, Japan) was used to purify IgG from the pooled sera of five RA patients with anti-cit-OPN antibodies and five healthy donors without anti-cit-OPN antibodies, following the manufacturer’s instructions.

### Cell adhesion assay

FLSs were obtained from RA patients undergoing total joint replacement (knee, hip, and elbow), as previously described [20]. FLSs were cultured in Dulbecco’s modified Eagle’s medium (DMEM) (Sigma-Aldrich, St. Louis, MO, USA) containing 10% fetal bovine serum and penicillin/streptomycin. The 96-well plates were coated with 50 mL of 5 mg/mL OPN or ovalbumin (OVA, LIONEX GmbH, Braunschweig, Germany), incubated overnight at 4 °C, and washed twice with 350 mL of phosphate buffered saline (PBS). The wells were blocked with 200 mL/well of serum-free DMEM containing 1% bovine serum albumin, 1 mM CaCl_2_, and 1 mM MgCl_2_ for 60 min at room temperature. Wells were washed with 350 mL PBS and incubated with 100 mL ethylenediaminetetraacetic acid-detached fibroblast-like synoviocytes (FLSs) (5 × 10^4^/well) in serum-free DMEM containing 1 mM CaCl_2_ and 1 mM MgCl_2_ for 15 min at room temperature with IgG from anti-cit-OPN-antibody-positive RA patients or healthy donors. The cells were incubated at 37 °C for 120 min. After washing, the number of cells was measured using the 3-(4,5-Dimethylthiazol-2-yl)-2,5-diphenyltetrazolium bromide (MTT) assay (Cayman Chemical, MI, USA), according to the manufacturer’s protocol.

### RNA preparation and quantitative real-time PCR (RT-qPCR)

RNA was extracted from using the RNeasy Mini Kit (Qiagen, Venlo, Netherlands), and cDNA was synthesized using ReverTra Ace qPCR RT Master Mix (TOYOBO, Osaka, Japan) according to the manufacturer’s protocol [21]. RT-qPCR was performed on a StepOnePlus system (Applied Biosystems, Foster City, CA, USA) using THUNDERBIRD® SYBR qPCR Mix (TOYOBO). Gene expression was calculated using the comparative ΔΔCt method and normalized to that of GAPDH, a housekeeping gene. Primer sequences are shown in Supplementary Table 1.

### Proliferation assay

FLSs (3 × 10^4^ cells/well) were cultured in 96-well plates with various concentrations of OPN for 7 days. MTT assay was performed as previously described.

### Mice

All animal studies were conducted in accordance with the principles of the Kyoto University Committee of Animal Resources, which is based on the International Guiding Principles for Biomedical Research Involving Animals. The experimental sample size was determined in our previous studies [21, 22]. All of the purchased mice were of a similar age and were randomly assigned to a treatment group; no randomization allocation sequence was assigned.

For the experimental arthritis model, cit-OPN (initially 8 mg, then 5.6 mg) was subcutaneously injected with complete Freund’s adjuvant in the tails of 6-week-old female DBA/1 mice (Clea-Japan, Fujinomiya, Japan), three times every 2 weeks. Arthritis was induced by intraperitoneal injection (75 ml) of KBxN serum [23]. For another arthritis model, 8-week-old female SKG mice (Clea-Japan, Fujinomiya, Japan) were injected subcutaneously with citrullinated osteopontin (initially 8 mg, then 5.6 mg, finally 4.8 mg) three times every 2 weeks with complete Freund’s adjuvant. Arthritis was induced by intraperitoneal injection of 20 mg of mannan (Sigma) [21]. The severity of arthritis was scored on a 3-point scale in a blinded fashion for each leg by two investigators, as previously described [21, 22]. The histological findings of each ankle joint were quantified by a blinded observer based on the degree of synovitis, as described previously [24].

### Statistical analysis

Statistical analyses were performed using the JMP Pro 16 software (SAS Institute Inc., Cary, NC, USA) and GraphPad Prism 7 (GraphPad Software, San Diego, CA, USA). Two-tailed unpaired *t*-tests were used to compare the categorical data. Holm–Sidak’s multiple comparison test was used to compare three or more groups. Spearman’s rank correlation coefficient was used to measure the association between the anti-cit-OPN and anti-CCP antibody levels. The survival of patients treated with TNF inhibitor and cytotoxic T lymphocyte-associated antigen-4-Ig (CTLA4-Ig) was examined using the Kaplan–Meier method and compared statistically using a stratified log-rank test. The termination of treatment because of lack of efficacy was considered the endpoint. Data are expressed as mean ± standard error of the mean (SEM) unless otherwise noted. Statistical significance was set at *p* less than 0.05. In all experiments, * *p* < 0.05, ** *p* < 0.01, and *** *p* < 0.001.

## Results

### cit-OPN is an antigen recognized by RA patients’ sera

**Fig. 1:**
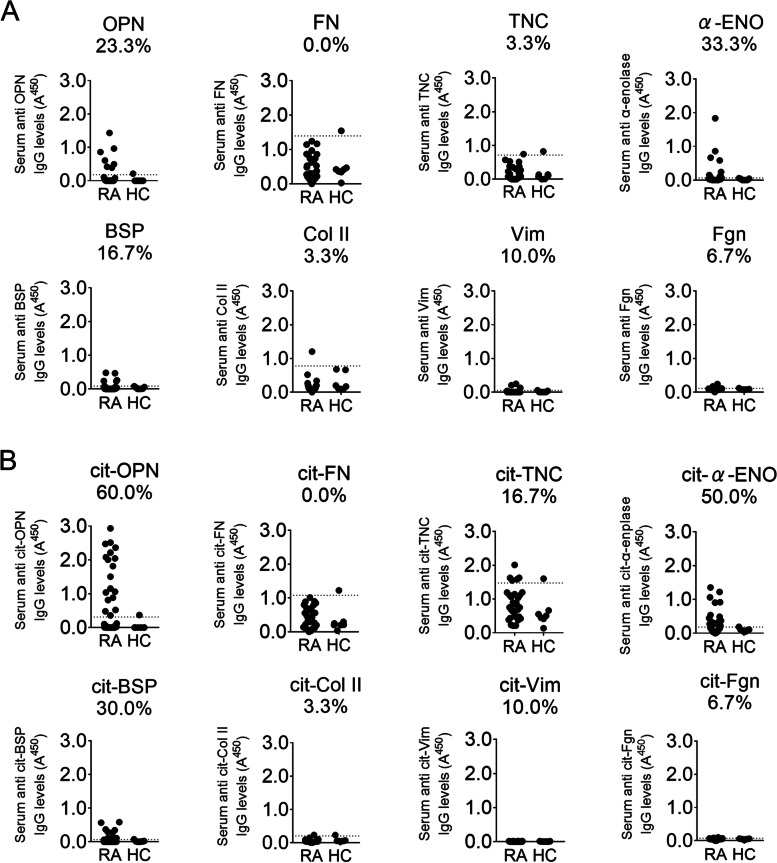
Positivity of autoantigen in sera of patients with rheumatoid arthritis (RA). **A** Serum IgG antibodies against osteopontin (OPN), fibronectin (FN), tenascin-C (TNC), α-enolase (α-ENO), bone sialoprotein (BSP), type 2 collagen (Col II), vimentin (Vim), and fibrinogen (Fgn) were quantified by enzyme-linked immunosorbent assay (ELISA). **B** Antibodies against citrullinated osteopontin (cit-OPN), citrullinated fibronectin (cit-FN), citrullinated tenascin-C (cit-TNC), citrullinated α-enolase (cit-α-ENO), citrullinated bone sialoprotein (cit-BSP), citrullinated type 2 collagen (cit-Col II), citrullinated vimentin (cit-Vim), and citrullinated fibrinogen (cit-Fgn) were tested. Serum samples from 30 RA patients and 7 healthy donors were used. The dashed line indicates the cutoff, defined as the mean plus two standard deviations (SDs), and the positivity of each antibody in RA patients is shown

Extracellular matrix glycoproteins, including fibronectin, tenascin-C, type II collagen, vimentin, fibrinogen, and OPN, are recognized by autoantibodies in RA patients. First, we tested the binding reactivity of IgG to extracellular matrix proteins using serum samples from 30 patients with RA and seven healthy controls by ELISA. When the mean plus two standard deviations of optical density (OD) of sera from healthy donors was set as the threshold [19, 25], 23.3%, 0.0%, 3.3%, 33.3%, 16.7%, 3.3%, 10.0%, and 6.7% patients with RA had autoantibodies against OPN, fibronectin, tenascin-C, α-enolase, bone sialoprotein (BSP), type II collagen, vimentin, and fibrinogen, respectively (Fig. 1A). Increased reactivity was observed in a larger proportion of patients when these proteins were citrullinated. Intriguingly, 60.0% of RA patients were positive for anti-cit-OPN antibodies, while 0.0%, 16.7%, 50.0%, 30.0%, 3.3%, 10.0%, and 6.7% of RA patients had antibodies against citrullinated fibronectin, tenascin-C, α-enolase, BSP, type II collagen, vimentin, and fibrinogen, respectively (Fig. 1B). We also tested several recombinant OPN proteins from different manufactures and cell lines. We found the positivity of the autoantibody was different among the proteins and determined to use the one with highest positivity (Supplementary Fig. 1). Since higher positivity in patients was found with anti-cit-OPN antibodies, we decided to investigate the role of anti-cit-OPN antibodies in RA.

### IgG from RA patients’ sera with anti-cit-OPN antibody increased the binding of FLSs with OPN

**Fig. 2:**
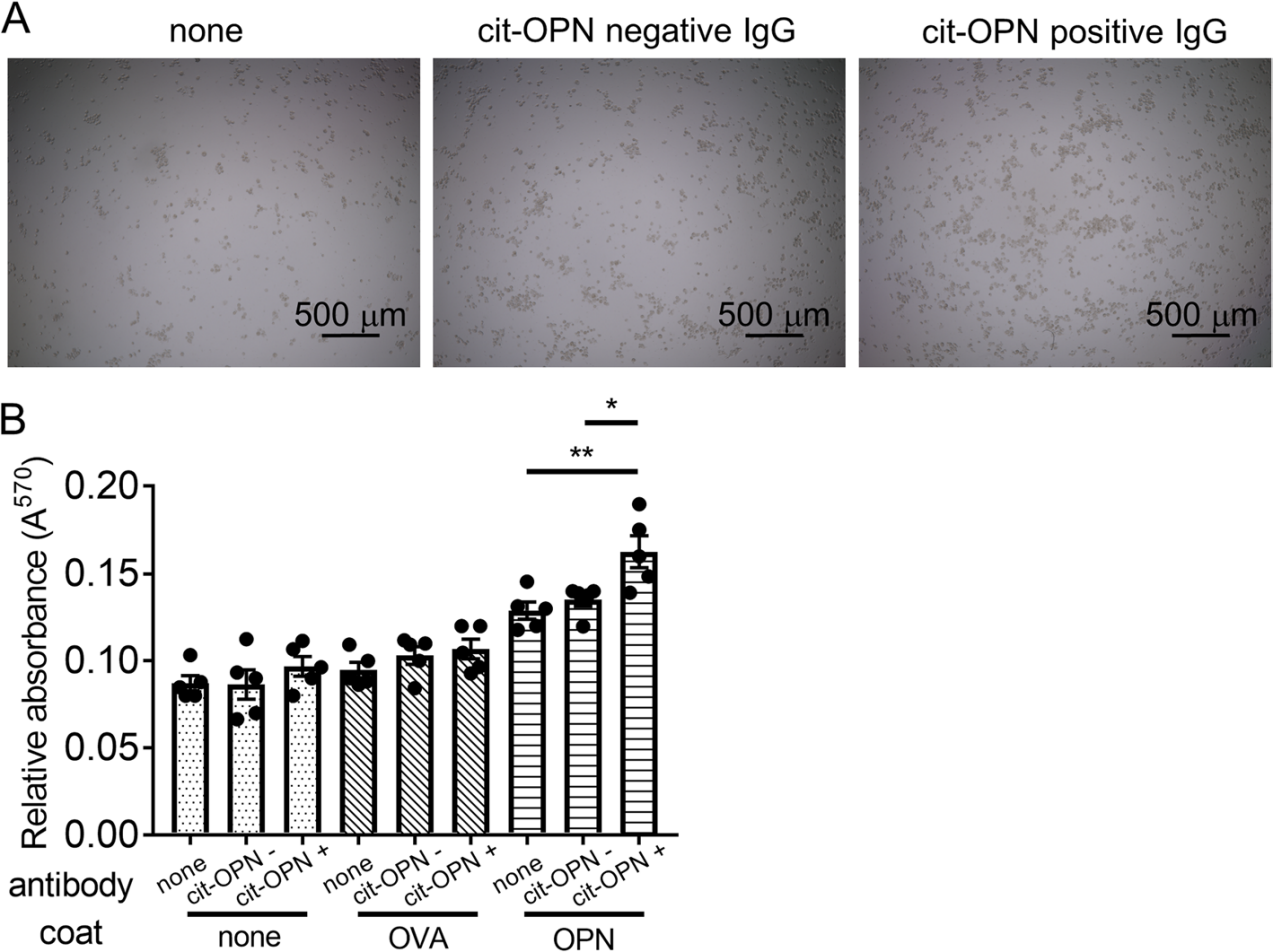
IgG from RA patients’ sera with anti-cit-OPN autoantibodies enhanced binding of fibroblast-like synoviocyte (FLSs) with OPN. **A** The 96-well plates were coated with OPN. FLSs were transferred to protein-coated plates and incubated at 37 °C for 120 min with pooled IgG from anti-cit-OPN positive RA patients or anti-cit-OPN negative RA patients. After washing, the number of cells was estimated using a 3-(4,5-Dimethylthiazol-2-yl)-2,5-diphenyltetrazolium bromide (MTT) assay. **B** Quantification of the five different donors. Data are shown as mean ± SEM from the aggregate data. * *p* < 0.05 and ** *p* < 0.01 by Tukey–Kramer test

The adhesion of FLSs to immobilized OPN was tested. An increased number of FLSs were bound to OPN compared to ovalbumin (Fig. 2A, B). IgG from RA patients’ serum with anti-cit-OPN antibody increased the binding of FLSs with OPN compared with IgG from those without anti-cit-OPN antibody, whereas IgG from RA patients’ sera did not increase the binding of FLSs to OVA.

### OPN induced the production of IL-6 and MMP, and proliferation in TNF-stimulated FLSs

**Fig. 3:**
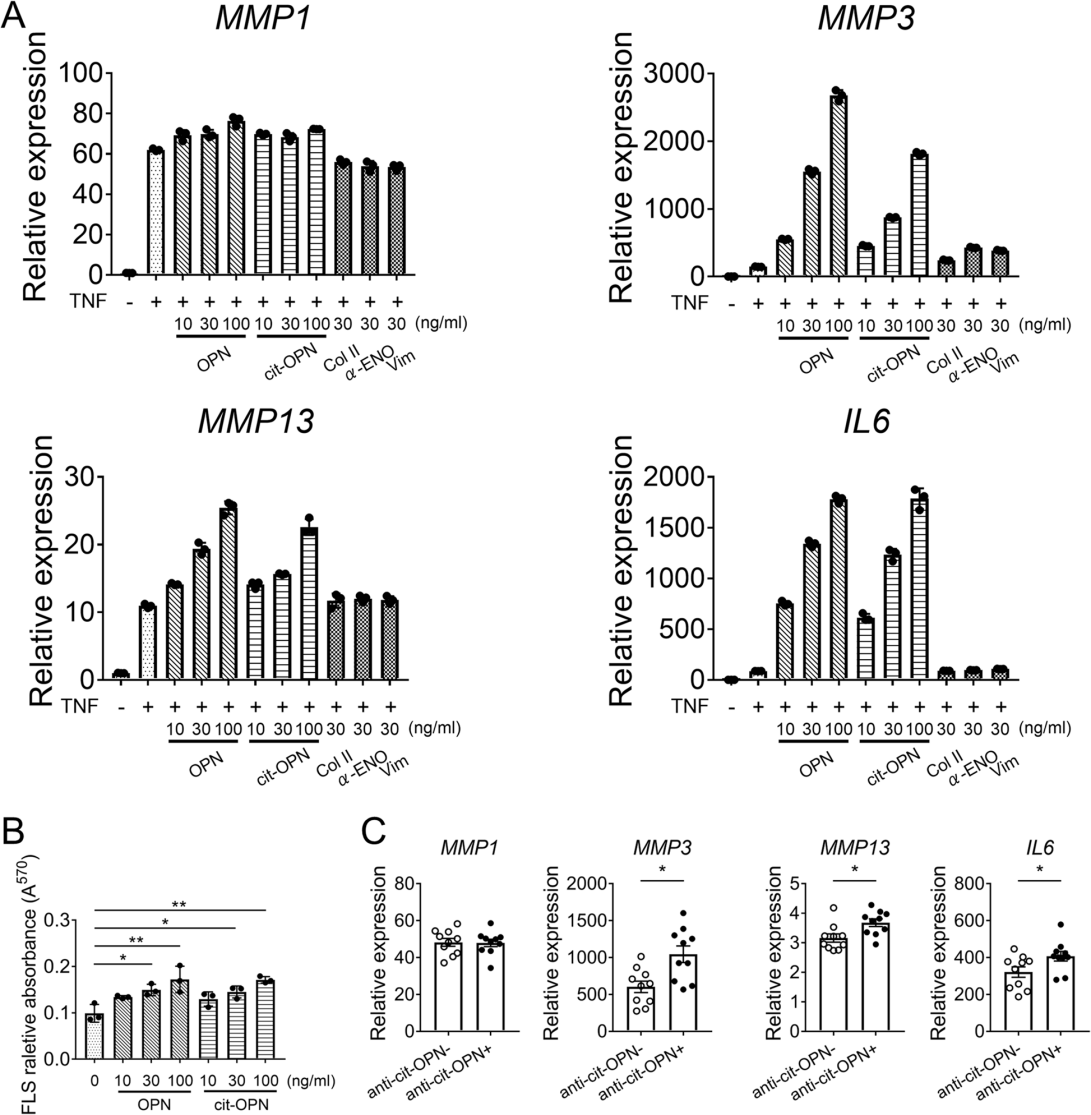
OPN induced IL-6 and MMP production in TNF-stimulated FLSs and IgG from patients with anti-OPN antibody increased their production. **A** RT-qPCR analysis of inflammatory genes (normalized relative to GAPDH mRNA). FLSs were stimulated with TNF (5 ng/ml) and OPN, cit-OPN, Col II, α-ENO, or Vim for 24 h. Data represent the mean ± SEM of triplicates from one representative experiment of three independent donors. **B** Proliferation of FLSs. The FLSs were incubated with OPN or cit-OPN for 1 week. The number of cells was quantified using the MTT assay. Results are presented as the mean ± SD from three independent donors. **C** FLSs were stimulated with TNF (5 ng/ml) and OPN for 24 h. IgG from anti-cit-OPN-positive RA patients or anti-cit-OPN-negative RA patients were added simultaneously. Data represent the mean ± SEM of 10 independent donors. Data are shown as the mean ± SEM. * *p* < 0.05 and ** *p* < 0.01 by Tukey–Kramer test (**B**) or Mann–Whitney’s *U* test (**C**)

To test whether recombinant OPN stimulates FLSs via integrin, we examined the levels of FAK phosphorylation. Immunoblot analysis showed that FAK phosphorylation was increased by OPN stimulation in FLSs (Supplementary Fig. 2). OPN mediates IL-6 production by FLSs during their interaction with B cells [26]. We confirmed that OPN induced not only IL-6 production in TNF-stimulated FLSs, but also MMP3 production (Fig. 3A and Supplementary Fig. 3). cit-OPN also induced IL-6 and MMP3, though the effect of cit-OPN was weaker than that of OPN. We also confirmed other autoantigens including α-enolase, type II collagen, or vimentin did not have such effect. In addition, OPN and cit-OPN promoted the proliferation of FLSs when added to the culture medium (Fig. 3B). Furthermore, FLSs were incubated with IgG from RA patients’ sera along with OPN and TNF. IgG from RA patients’ serum with anti-cit-OPN antibody increased the expressions of MMP3, MMP13, and IL-6 compared with IgG from RA patients without anti-cit-OPN antibody (Fig. 3C).

### Anti-cit-OPN antibodies aggravated inflammatory arthritis

In vitro experiments suggested that IgG with anti-cit-OPN antibodies increased OPN stimulation in FLSs by increasing OPN binding. To directly test whether anti-cit-OPN antibodies aggravate inflammatory arthritis, we immunized mice with cit-OPN and induced arthritis using two models.

**Fig. 4:**
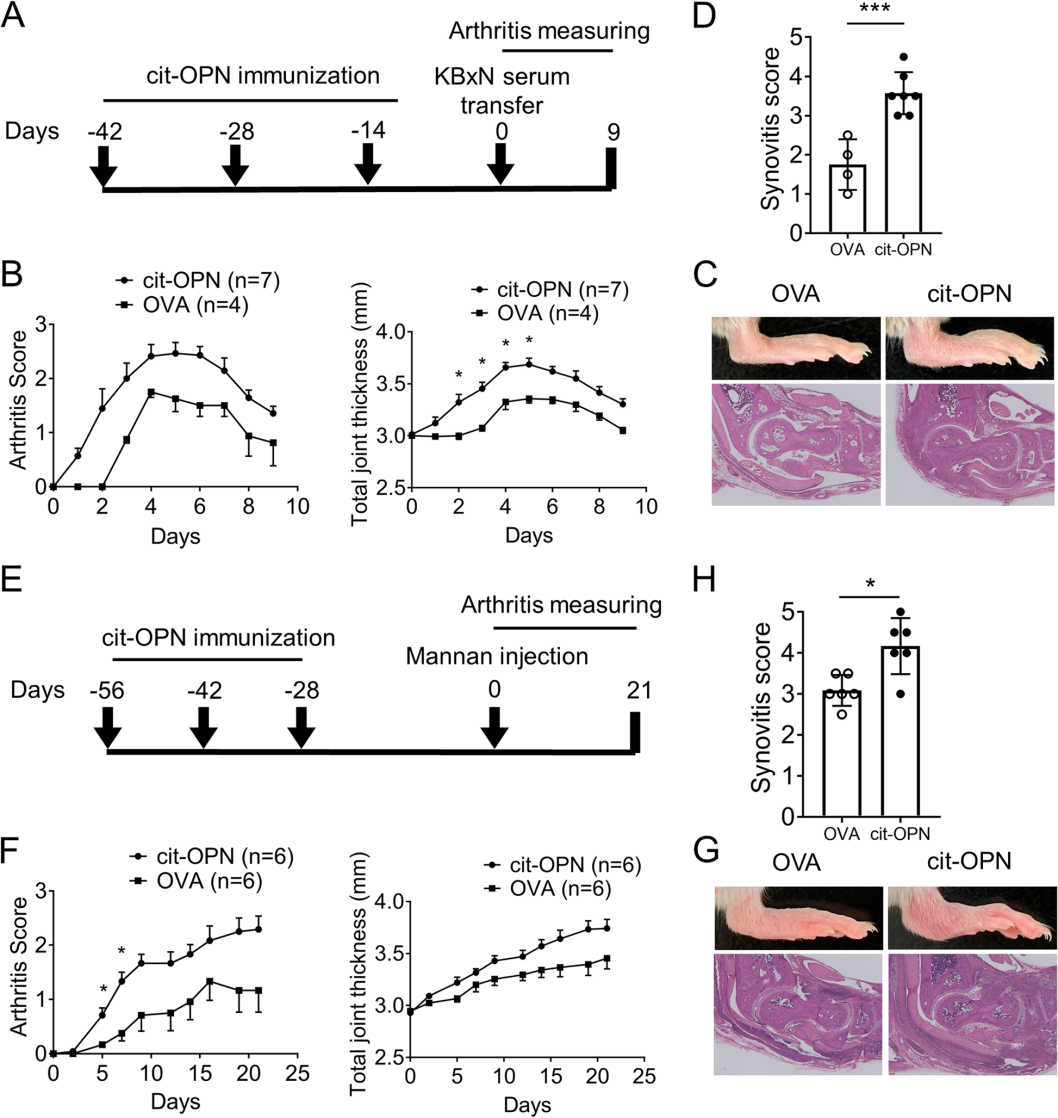
Anti-cit-OPN antibody aggravates inflammatory arthritis. **A** Arthritis in DBA/1 mice immunized with ovalbumin or citrullinated osteopontin was induced by intraperitoneal injection of KBxN serum. **B** Time course of changes in the arthritis severity score and joint swelling. **C**, **D** Histologic sections from the ankle stained with hematoxylin and eosin staining (**C**) and assessed for the histologic synovitis scores (**D**) (*n* = 4–7 from two independent experiments). **E** Arthritis in SKG mice immunized with ovalbumin or citrullinated osteopontin was induced by mannan. **F** Time course of changes in the arthritis severity score and joint swelling. **G**, **H** Histological sections from the ankle stained with hematoxylin and eosin (**G**) and assessed for histological synovitis scores (**H**) (*n* = 6, from two independent experiments). All data are shown as the mean ± SEM. * *p* < 0.05 and *** *p* < 0.001 by Holm–Sidak test (**B**, **F**) or Mann–Whitney’s *U* test (**D**, **H**)

First, we immunized DBA/1 mice with cit-OPN and induced arthritis using KBxN serum (Fig. 4A). We confirmed that anti-cit-OPN antibodies were successfully created in mice immunized with cit-OPN (Supplementary Fig. 4A) and that human OPN could not be detected in immunized mice sera (data not shown), which suggests that direct effect of OPN to arthritis was minimum. Immunization with cit-OPN alone did not induce arthritis (data not shown). Mice with the anti-cit-OPN antibody treated with KBxN serum exhibited severe arthritis compared to mice without the anti-cit-OPN antibody (Fig. 4B–D). We also tested the effect of the anti-cit-OPN antibodies using mannan-induced arthritis in ZAP70-mutated SKG mice, which developed T cell-mediated autoimmune arthritis (Fig. 4E, Supplementary Fig. 4B). SKG mice immunized with cit-OPN developed more severe arthritis than mice without cit-OPN (Fig. 4F–H).

### Anti-cit-OPN antibody in RA patients affects the survival rate of anti-rheumatic drugs

**Fig. 5:**
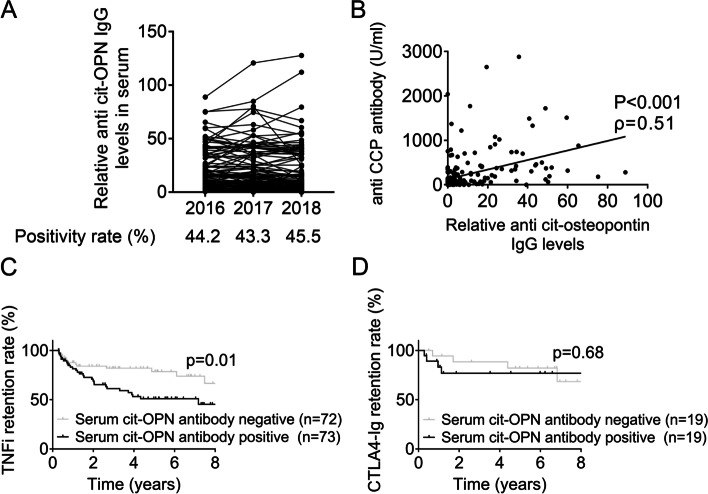
Survival rate of anti-rheumatic drugs between RA patients with and without anti-cit-OPN antibodies. **A** Positivity of anti-cit-OPN antibody from RA sera collected over 3 consecutive years, measured by ELISA. **B** Relationship between serum anti-CCP antibody and anti-cit-OPN antibody using stored serum in the 2016 cohort. **C** Survival rate of TNF inhibitors due to lack of efficacy between RA patients with and without anti-cit-OPN antibodies in the 2016 cohort. Patients in whom TNF inhibitors were discontinued within 90 days were excluded. **D** Survival rate of CTLA4-Ig due to lack of efficacy between RA patients with and without anti-cit-OPN antibodies. Patients in whom the drug was discontinued within 90 days were excluded

The characteristics of anti-cit-OPN antibody-positive RA patients were investigated using the sera of 224 RA patients collected cross-sectionally during the annual survey of the KURAMA cohort for consecutive 3 years starting in 2016. Anti-cit-OPN antibody was positive in 44.2, 43.3, and 45.5% of patients in the 2016, 2017, and 2018 cohorts, respectively, whereas 77.8, 77.7, and 78.0% of patients were positive for the CCP2 assay in the 2016, 2017, and 2018 cohorts (Table 1). No increase in positivity was observed over time (Fig. 5A). A significant correlation was found between the anti-CCP2 and anti-cit-OPN antibody titres (Fig. 5B). Approximately 50% of the patients were treated with biological DMARDs (Table 1). Interestingly, the survival rate of TNF inhibitors was significantly lower in patients with anti-cit-OPN antibodies than in those without it, when the reason of discontinuation was lack of effectiveness. In contrast, the presence or absence of cit-OPN antibody did not affect the retention rate of CTLA-4 Ig (Abatacept) (Fig. 5C).

**Table 1:** Clinical characteristics of the study population in the 2016–2018 KURAMA cohort

*n* = 224	2016	2017	2018
The positivity of anti-cit-OPN antibody, %	44.2	43.3	45.5
The positive rate of anti-cit-OPN antibody in anti-CCP antibody-positive patients, %	53.5	50.9	52.4
DAS28-CRP	2.09 ± 0.81	2.10 ± 0.81	1.94 ± 0.73
DAS28-ESR	2.64 ± 0.96	2.66 ± 0.99	2.43 ± 0.97
Anti-CCP antibody, U/mL	266.0 ± 444.7	355.6 ± 566.3	403.5 ± 772.1
The rate of anti-CCP antibody positive, %	77.8	77.7	78.0
Rheumatoid factor, IU/mL	119.7 ± 240.4	120.6 ± 264.4	130.1 ± 296.0
The rate of rheumatoid factor positive, %	77.7	80.0	78.6
CRP, mg/dL	0.36 ± 0.74	0.36 ± 0.64	0.38 ± 0.73
ESR 1 h, mm/h	20.6 ± 16.3	21.1 ± 16.7	20.3 ± 17.6
ptVAS, 0–100 mm	22.3 ± 21.8	22.9 ± 21.8	23.0 ± 22.6
The use of PSL, %	22.8	22.3	22.3
PSL dose, mg/day	3.78 ± 4.16	3.29 ± 2.10	3.31 ± 1.73
The use of MTX, %	75.0	74.6	73.7
MTX dose, mg/week	7.37 ± 3.13	7.50 ± 3.16	7.47 ± 3.28
The use of biologic DMARDs, %	50.9	52.2	54.5

*CRP* C-reactive protein, *C* reactive protein, *ESR* Erythrocyte sedimentation rate, *CCP* Cyclic citrullinated peptide, *VAS* Visual analog scale, *PSL* Prednisolone, *MTX* Methotrexate, *DMARDs* Disease-modifying anti-rheumatic drugs

## Discussion

In this study, we demonstrated that RA patients with anti-cit-OPN antibodies were relatively common. These antibodies enhanced the adhesion between OPN and FLSs, increased the expression of inflammatory cytokines including IL-6, aggravated arthritis, and lowered the retention rate of TNF inhibitors in RA patients.

OPN plays a role in the regulation of immune responses at multiple levels [27]. This protein is complex to analyse, since it binds to a series of different integrins through RGD, SVVYGLR, and RSKSKKFRR sequences. In addition to its secreted forms, an intracellular form of OPN mediates aspects of intracellular signalling. OPN produced by dendritic cells supports IL-17 expression in Th17 cells. It mediates the development of germinal centers and immunoglobulin production in B cells. Regarding the relationship between OPN and RA, OPN-deficient mice are protected from inflammatory arthritis [27]. Recent single-cell RNA-sequence analysis has revealed that SPP1(OPN)-positive macrophages are associated with active RA [28].

In the past, there have been attempts to treat RA using anti-OPN neutralizing antibodies because OPN plays a pivotal role in the pathogenesis of RA. One anti-OPN monoclonal antibody, 23C3, was found effective in inhibiting the development of anti-type II collagen antibody-induced arthritis thorough suppressing the T cell response [29]. Another antibody, M5, recognizing SLAYGLR/SVVYGLR sequence, abrogated monocyte migration toward the thrombin-cleaved form of OPN. It also provided protection in both mouse and non-human primate models of RA, which is mediated by α9 integrin binding [27]. These antibodies to treat RA possibly block the interaction of OPN and integrin, and they need be distinguished from autoantibody in this study.

Human OPN contains 10 arginine residues, which are potential target of citrullination, at full length. Furthermore, OPN is cleaved not only by thrombin but also by MMP3, MMP7, and MMP9, indicating that various forms of OPN exist in human tissues [30]. For this reason, it was not easy to prepare monoclonal or polyclonal antibodies against pathological cit-OPN and isolate cit-OPN from the synovial tissue of RA patients. The citrullinated arginine in vivo and the distribution of citrullinated OPN in the tissues of patients with RA should be determined in the future.

Information regarding the role of autoantibodies against each antigen in RA is limited. The presence of ACPAs in patients with more destructive RA and the accumulation of citrullinated peptides in rheumatoid joints suggest a possible role of ACPAs in RA pathogenesis [31]. It includes activation of macrophages via immune complex formation and agonistic activity, and promotion of NETosis by ACPA-forming ICs, which releases massive amounts of citrullinated antigen to drive ACPA production, fibroblast migration, and osteoclast differentiation [31–33]. However, the mechanisms by which each autoantibody specifically affects the joint and bone compartments remain unclear. Though previous studies have used antibodies isolated from pooled serum, monoclonal antibodies against citrullinated fibrinogen, citrullinated vimentin, and ACPA-reactive monoclonal antibodies isolated from RA patients, to the best of our knowledge, there are no reports that ACPAs modulate protein binding or contribute to protein stability. Anti-cit-OPN antibodies have yet to be studied in detail. This study is valuable for clarifying the role of anti-cit-OPN antibodies in RA.

Several studies have examined the association between the presence of autoantibodies and the RA phenotype using peptide or protein arrays. Van Beers et al. reported that the clinical characteristics at baseline or disease progression in patients with early RA did not correlate with the reactivity to each antigen of the ACPA using 20 citrullinated peptides with 374 early RA patients [34]. Another study examined the reactivity of autoantibodies to 36 epitopes of 10 antigens in 1006 RA patients, but only found an association between autoantibodies to fibronectin and obesity, and between autoantibodies to fibrinogen and pneumonopathy [35]. The target proteins/peptides examined in these reports were α-enolase, fibrinogen, fibronectin, filaggrin, histones, vimentin, collagen type II, apolipoprotein A-I, and apolipoprotein E, but neither OPN nor cit-OPN were mapped on the array.

The strength of this study is the use of the OPN protein rather than the OPN peptide to detect autoantibodies. The difference in the positivity against the recombinant OPN proteins (Supplementary Fig. 1) suggests the importance of the three-dimensional structure of the antigen. This allowed us to detect OPN and anti-cit-OPN antibodies with higher sensitivity than in previous reports and identify their roles in RA progression [14].

Interestingly, RA patients with anti-cit-OPN antibodies have a lower retention rate of TNF inhibitors than those without, and the presence of anti-cit-OPN antibodies did not affect the retention rate of CTLA4-Ig (Abatacept). CTLA4-Ig binds to the co-stimulatory molecules CD80 and CD86 on antigen-presenting cells (APC), thereby blocking their interaction with CD28 on T cells. It affects B cells by binding to CD80 and CD86 on the surface of B cells and inhibit the co-stimulation and activation of T cells, leading to downregulation of inflammatory mediators [36]. In patients with anti-cit-OPN antibodies, it is likely that B cells producing anti-cit-OPN aggravated arthritis, and treatments that target B cells were more effective than treatments that neutralize TNF or target TNF-producing cells.

The limitations of this study are that we were neither able to identify the binding partner of OPN nor a mechanism by which the presence of anti-cit-OPN antibodies enhances the binding of OPN to the receptor. The post-translational forms of OPN were diverse, and we could not clarify how cit-OPN was cleaved and modified in the synovial tissues. In addition, the binding sites of autoantibodies were also unknown because the variety of OPN forms makes it difficult to purify with a single antibody. This is also the case in mouse experiments. We immunized with cit-OPN, expecting that immunization with cit-OPN would produce more autoantibodies than that with OPN. It remains unclear whether the anti-cit-OPN antibodies produced in mice recognize cit-OPN or non-cit-OPN. If this stumbling block is removed, further insight into how anti-OPN antibodies exacerbate arthritis in RA can be clarified.

In conclusion, anti-cit-OPN autoantibodies were detected in approximately 40–50% of RA patient sera. The anti-cit-OPN antibody increased the binding of FLSs to OPN, thereby inducing the production of IL-6 and MMPs and proliferation in FLSs. Anti-cit-OPN antibodies aggravated inflammatory arthritis and reduced the retention rate of TNF inhibitors in patients with RA.

## Supplementary Information

**Additional file 1:** **Supplementary Fig. 1. **Reactivity of autoantigen in sera of patients with rheumatoid arthritis (RA) was tested against various recombinant osteopontin. Osteopontin (OPN)_R (R&D, 1433-OP-050) derives from NS0 mouse myeloid cell line. OPN_AC (ACROBiosystems, OPN-H5227) and OPN_AbH (Abcam, ab281819) derive from HEK293 cells. OPN_AbE (Abcam, ab92964) is produced using E. Coli. The result of OPN_R is identical of the upper left OPN result of Fig. 1. **Supplementary Fig. 2. **Immunoblot analysis of whole-cell lysates using FAK and pFAK antibodies. Fibroblast-like synoviocytes (FLSs) were stimulated with OPN (150 ng/ml) for 0–60 min. Blots show representative data (*n*=3), and densitometric quantification is shown. **Supplementary Fig. 3. **Inflammatory response of FLSs by TNF and OPN. FLSs were stimulated with TNF (5 ng/ml) and OPN for 24 h. Data represent the mean ± SEM of three independent donors. **Supplementary Fig. 4. **Antibody titer in serum of mice immunized with citrullinated osteopontin (cit-OPN). A In the model of DBA/1 mice immunized with ovalbumin (OVA) or cit-OPN and intraperitoneally administered with KBxN serum, the titre of ant-cit-OPN antibodies in serum was confirmed by ELISA. B In SKG mice immunized with ovalbumin or cit-OPN and treated with mannan, serum cit-OPN antibody titre was measured by ELISA. **Supplementary Table 1. **Sequence of Real-time PCR primers used in this study.
