# Supplementary material for: Identification of anti-citrullinated osteopontin antibodies and increased inflammatory response by enhancement of osteopontin binding to fibroblast-like synoviocytes in rheumatoid arthritis
Source: Arthritis Res Ther. 2023 Feb 17;25:25. doi: 10.1186/s13075-023-03007-9 (PMC9936655; doi:10.1186/s13075-023-03007-9)
Supplement: Supplementary file 1 — Additional file 1: Supplementary Fig. 1. Reactivity of autoantigen in sera of patients with rheumatoid arthritis (RA) was tested against various recombinant osteopontin. Osteopontin (OPN)_R (R&D, 1433-OP-050) derives from NS0 mouse myeloid cell line. OPN_AC (ACROBiosystems, OPN-H5227) and OPN_AbH (Abcam, ab281819) derive from HEK293 cells. OPN_AbE (Abcam, ab92964) is produced using E. Coli. The result of OPN_R is identical of the upper left OPN result of Fig. 1. Supplementary Fig. 2. Immunoblot analysis of whole-cell lysates using FAK and pFAK antibodies. Fibroblast-like synoviocytes (FLSs) were stimulated with OPN (150 ng/ml) for 0–60 min. Blots show representative data (n=3), and densitometric quantification is shown. Supplementary Fig. 3. Inflammatory response of FLSs by TNF and OPN. FLSs were stimulated with TNF (5 ng/ml) and OPN for 24 h. Data represent the mean ± SEM of three independent donors. Supplementary Fig. 4. Antibody titer in serum of mice immunized with citrullinated osteopontin (cit-OPN). A In the model of DBA/1 mice immunized with ovalbumin (OVA) or cit-OPN and intraperitoneally administered with KBxN serum, the titre of ant-cit-OPN antibodies in serum was confirmed by ELISA. B In SKG mice immunized with ovalbumin or cit-OPN and treated with mannan, serum cit-OPN antibody titre was measured by ELISA. Supplementary Table 1. Sequence of Real-time PCR primers used in this study. [file 13075_2023_3007_MOESM1_ESM.docx]

**Supplemental information**

**Anti-citrullinated osteopontin antibody enhances the binding of osteopontin to synovial cells and aggravates rheumatoid arthritis**

Umemoto et al.

Table of contents

Supplementary Fig. 1 Page 2

Supplementary Fig. 2 Page 3

Supplementary Fig. 3 Page 4

Supplementary Fig. 4 Page 5

Supplementary Table 1 Page 6

**
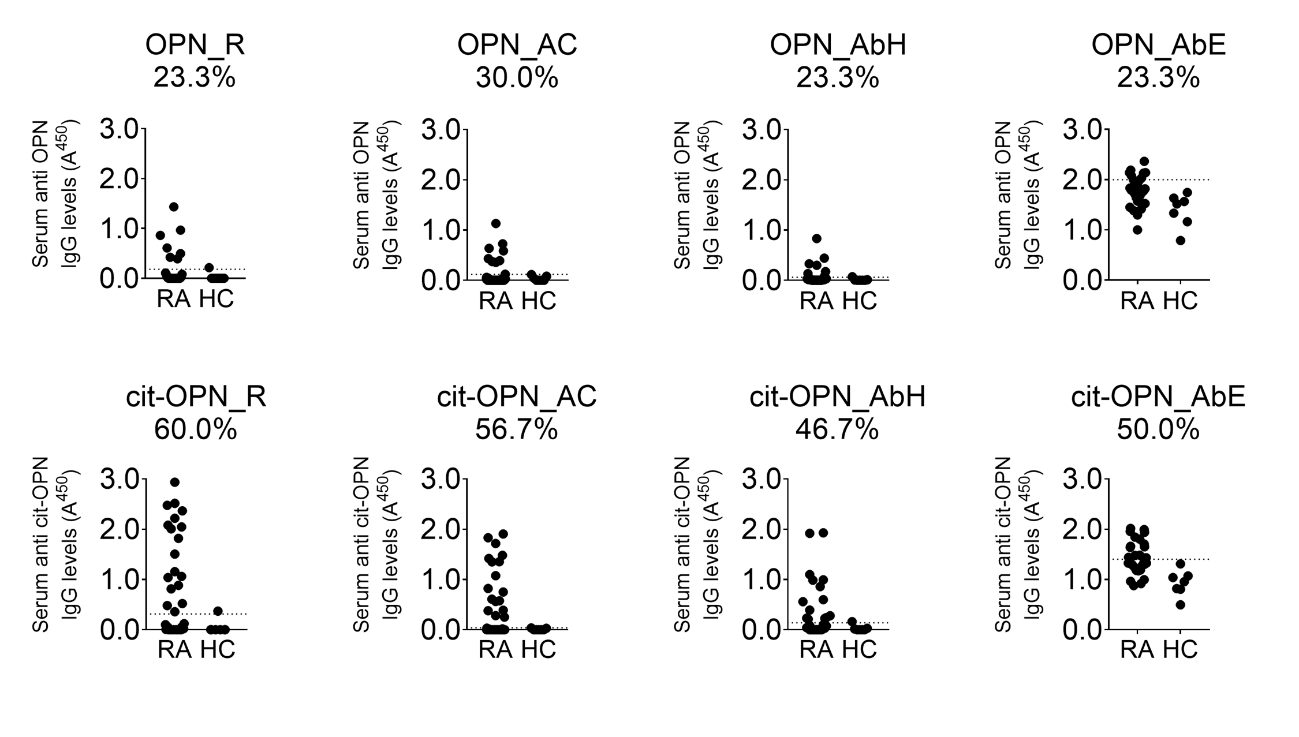
**

**Supplementary Fig. 1 Reactivity of autoantigen in sera of patients with rheumatoid arthritis (RA) was tested against various recombinant osteopontin.**

Osteopontin (OPN)_R (R&D, 1433-OP-050) derives from NS0 mouse myeloid cell line. OPN_AC (ACROBiosystems, OPN-H5227) and OPN_AbH (Abcam, ab281819) derive from HEK293 cells. OPN_AbE (Abcam, ab92964) is produced using E. Coli. The result of OPN_R is identical of the upper left OPN result of Fig.1.

**
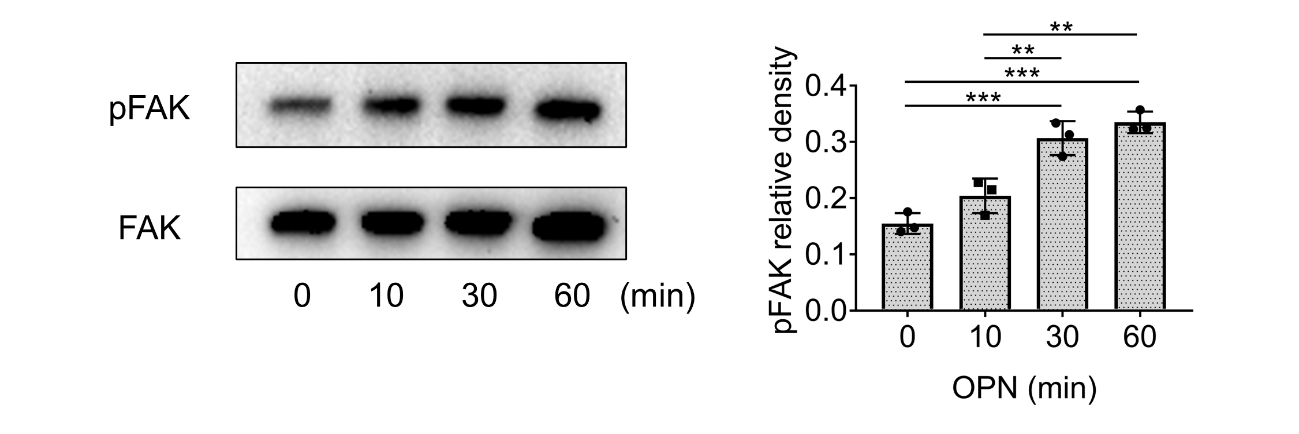
**

**Supplementary Fig. 2 Immunoblot analysis of whole-cell lysates using FAK and pFAK antibodies.** Fibroblast-like synoviocytes (FLSs) were stimulated with OPN (150 ng/ml) for 0–60 min. Blots show representative data (n=3), and densitometric quantification is shown.


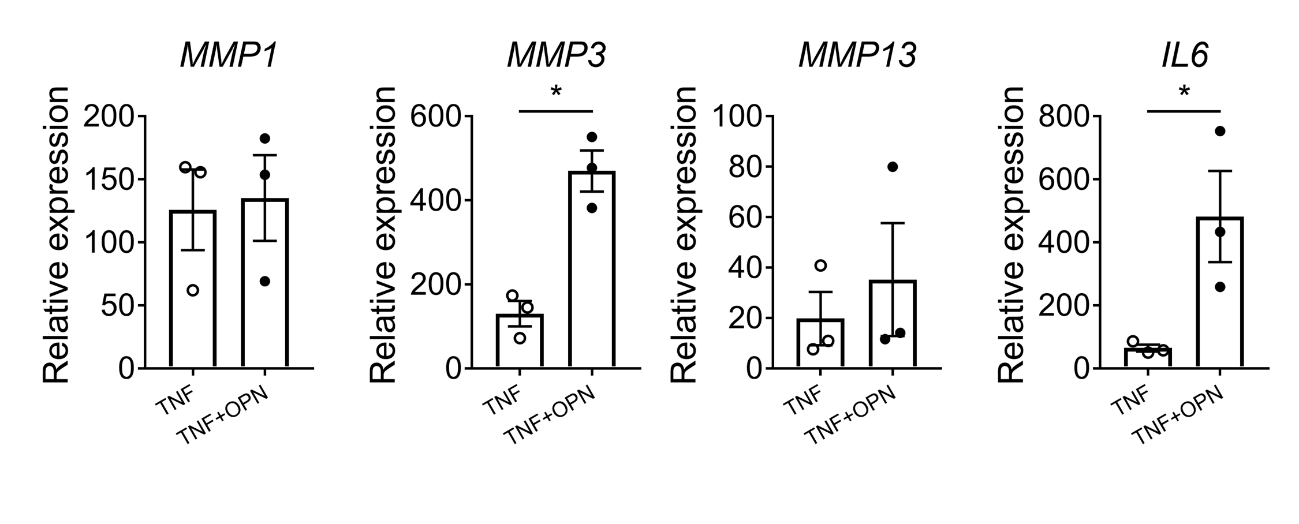


**Supplementary Fig. 3 Inflammatory response of FLSs by TNF and OPN.** FLSs were stimulated with TNF (5 ng/ml) and OPN for 24 h. Data represent the mean ± SEM of three independent donors.


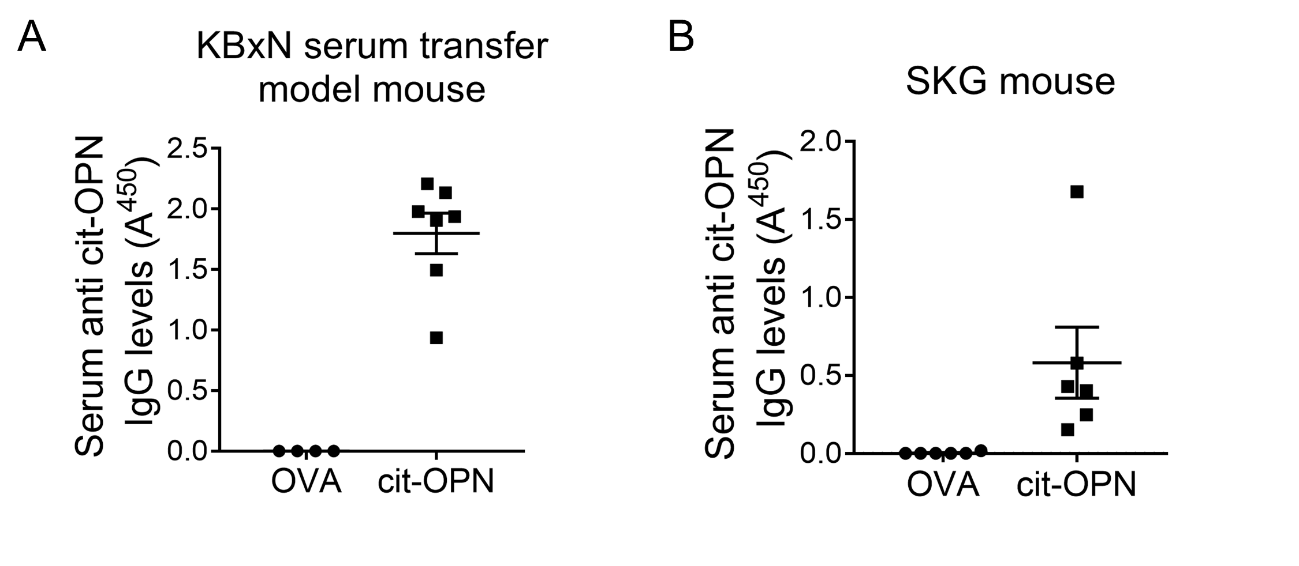


**Supplementary Fig. 4 Antibody titer in serum of mice immunized with citrullinated osteopontin (cit-OPN).**

**A** In the model of DBA/1 mice immunized with ovalbumin (OVA) or cit-OPN and intraperitoneally administered with KBxN serum, the titre of ant-cit-OPN antibodies in serum was confirmed by ELISA.

**B** In SKG mice immunized with ovalbumin or cit-OPN and treated with mannan, serum cit-OPN antibody titre was measured by ELISA.

**Supplementary Table 1. Sequence of Real-time PCR primers used in this study.**

| Primers | Sequences |
| --- | --- |
| MMP1 Fwd | GGGAGATCATCGGGACAACTC |
| MMP1 Rev | GGGCCTGGTTGAAAAGCAT |
| MMP3 Fwd | AGGTTTCCCTCCAACCGTGAG |
| MMP3 Rev | AGCCTGGCTCCATGGAATTTCT |
| MMP13 Fwd | ATCCAAAAACGCCAGACAAATG |
| MMP13 Rev | ATGCAGGCGCCAGAAGAATC |
| IL6 Fwd | TAATGGGCATTCCTTCTTCT |
| IL6 Rev | TGTCCTAACGCTCATACTTTT |
| GAPDH Fwd | ATCAAGAAGGTGGTGAAGCA |
| GAPDH Rev | GTCGCTGTTGAAGTCAGAGGA |
